# Stereotactic body radiation therapy for prostate cancer: a study comparing 3-year genitourinary toxicity between CyberKnife and volumetric-modulated arc therapy by propensity score analysis

**DOI:** 10.1186/s13014-023-02233-4

**Published:** 2023-02-23

**Authors:** Makoto Ito, Yasuo Yoshioka, Yuuki Takase, Junji Suzuki, Hironori Takahashi, Yoshitaka Minami, Ami Sakuragi, Yukihiko Oshima, Takahito Okuda, Kojiro Suzuki

**Affiliations:** 1 Department of Radiology, Aichi Medical University Hospital, 1-1 Yazako-Karimata, Nagakute, Aichi 480-1195 Japan; 2 Department of Radiation Oncology, Cancer Institute Hospital, Japanese Foundation for Cancer Research, 3-8-31 Ariake, Koto-Ku, Tokyo, 135-8550 Japan; 3 Department of Radiation Oncology, Toyota Memorial Hospital, 1-1-1 Heiwa-Cho, Toyota, Aichi 471-8513 Japan; 4 Department of Radiology, Nagoya University Hospital, 65 Tsurumai-Cho, Showa-Ku, Nagoya, Aichi 466-8560 Japan; 5 Department of Radiotherapy Quality Management Group, Toyota Memorial Hospital, 1-1-1 Heiwa-Cho, Toyota, Aichi 471-8513 Japan; 6 Department of Central Radiology, Aichi Medical University Hospital, 1-1 Yazako-Karimata, Nagakute, Aichi 480-1195 Japan

**Keywords:** Prostate cancer, SBRT, CyberKnife, VMAT

## Abstract

**Background:**

To investigate whether the rate of stereotactic body radiation therapy-related (SBRT-related) genitourinary (GU) toxicity is lower in patients with prostate cancer treated with CyberKnife.

**Methods:**

We retrospectively reviewed the medical records of patients with nonmetastatic prostate cancer at two institutions between 2017 and 2020. We analyzed 70 patients who were extracted by propensity score matching based on age, pre-treatment International Prostate Symptom Score (IPSS), and prostate volume. The patients were treated with SBRT, with a total dose of 36.25 Gy in five fractions over five consecutive weekdays, using CyberKnife or volumetric-modulated arc therapy (VMAT).

**Results:**

The low-, medium-, and high-risk patients were 2, 19, and 14, respectively, in the CyberKnife group and 4, 17, and 14, respectively, in the VMAT group. The median follow-up time in both groups was 3 years. One patient with CyberKnife died of unrelated causes. No biochemical or clinical recurrence, distant metastases, or death from prostate cancer was observed.

The peak values of IPSS in the acute phase (< 3 months) were significantly lower in the CyberKnife than in the VMAT group (CyberKnife:16.2 vs VMAT:20.2, p = 0.025). In multiple regression analyses, the treatment modality (p = 0.03), age (p = 0.01), bladder medication pre-irradiation (p = 0.03), and neoadjuvant androgen deprivation therapy (p = 0.04) contributed to the peak value of the acute-phase IPSS. The incidence of treatment-related grade 2 acute GU toxicity tended to be lower in the CyberKnife than the VMAT group (CyberKnife: 22.9% vs. VMAT: 45.7%, p = 0.077). No difference was noted between the groups with regard to late IPSS or GU toxicity and gastrointestinal toxicity in all phases. Toxicities of grade ≥ 3 have not been observed to date.

**Conclusions:**

Regardless of treatment modality, SBRT is effective in treating prostate cancer without serious toxicity. However, CyberKnife has an advantage over VMAT in terms of acute prostate symptoms.

## Background

According to the latest American Cancer Society report, the estimated number of new cases of prostate cancer by 2022 is 268,490 (27%), which makes it the most prevalent cancer among men [1]. Radiotherapy is a typical curative treatment for prostate cancer in the localized stage, other than prostatectomy. It is rich in variety and is broadly divided into external beam radiotherapy and brachytherapy [2]. Majority of the patients receive external beam radiotherapy, and stereotactic body radiation therapy (SBRT) has received particular attention in recent years. In recent clinical trials, SBRT has shown outcomes comparable to those of the conventional methods [3, 4]. In addition, SBRT offers clear logistic and cost benefits to patients and resource-utilization benefits to the healthcare system compared to other longer radiotherapy courses [5].

Two main treatment modalities are used for SBRT for prostate cancer: CyberKnife and linear accelerator. The CyberKnife system has the inherent geometrical targeting precision of a commercial arm-based robotic system carrying a compact X-band linear accelerator and integrated with radiographic imaging and visualization feedback systems. On the other hand, the linear accelerator is equipped with a multileaf collimator and delivers radiation precisely to the target, mainly using the volumetric-modulated arc therapy (VMAT) technique. The dose-physical characteristics of both the modalities are different [6]. Several studies for prostate cancer have reported that treatment planning with both modalities results in different dose distributions to the target and normal tissues [7, 8]. This may potentially cause differences in the clinical outcomes. However, the clinical results of SBRT for prostate cancer in many cases have been reported using either CyberKnife or VMAT [9, 10]. A few clinical trials have used both modalities, but their clinical differences remain unclear [4, 11].

In this study, we have compared the clinical outcomes of patients with prostate cancer who underwent SBRT using CyberKnife and VMAT. For genitourinary (GU) toxicity, we reviewed patient-reported outcomes and physician-recorded toxicities adjusted for background factors using propensity score matching.

## Methods

### Patients

We retrospectively reviewed the medical records of patients with nonmetastatic prostate cancer (cT1–T3a, N0, M0) at two institutions, between June 2017 and December 2020. Patients were treated with the CyberKnife M6 system (Accuray Inc., Sunnyvale, CA, USA) at Toyota Memorial Hospital or with VMAT (TrueBeam STx, Varian Medical Systems, Palo Alto, CA, USA) at Aichi Medical University Hospital. We included patients aged ≥ 20 years who had been treated with SBRT with radical intent. Of the 148 consecutive patients who met the criteria, six were excluded because of a short follow-up duration (< 1 year). Of the remaining 142 patients, 104 underwent treatment using CyberKnife and 38 using VMAT. Finally, we analyzed 70 patients who were extracted by propensity score matching. Matching was based on three factors reported to contribute to GU toxicity: age, pre-treatment International Prostate Symptom Score (IPSS), and prostate volume [12].

All patients underwent magnetic resonance imaging (MRI) of the pelvis and technetium-99 m-methylene diphosphonate bone scan for staging. Patients were classified into risk groups according to the National Comprehensive Cancer Network guidelines [13]. Radiotherapy alone was administered to low-risk patients (clinical stage T1–T2a, prostate-specific antigen [PSA] < 10 ng/mL, and Gleason score of 6). In contrast, intermediate-risk patients (clinical stage T2b–T2c, PSA = 10–20 ng/mL, and/or Gleason score of 7) received additional neoadjuvant androgen deprivation therapy (ADT) for 6 months. High-risk patients (clinical stage T3a, PSA > 20 ng/mL, and/or Gleason score ≥ 8) received additional neoadjuvant and adjuvant ADT for 2 years.

This retrospective study was approved by the ethics committees of the two institutions (Application No. R210-1, 2021-017), and the need for informed consent was waived.

### Radiotherapy

#### General procedures

Patients were immobilized in the supine position on a vacuum-formable mattress and administered a total dose of 36.25 Gy in five fractions. Irradiation was performed on five consecutive weekdays. However, two patients in the CyberKnife group underwent alternate-day treatment owning to patient preference, and one of them was included in the final analysis. Targets were contoured via registration of T2-weighted MRI sequences with planning computed tomography (CT) scans. The gross tumor volume (GTV) was defined as the entire prostate gland in low-risk patients. For those at intermediate and high risk, 1 cm of the proximal seminal vesicle was also included in the GTV. The prescription dose was defined as the D95 of the planning target volume (PTV). The patient was trained for pelvic reproduction at the time of the first consultation. We provided dietary guidance and prescribed laxatives and antifoaming agents such as dimethicone according to the condition of the gastrointestinal (GI) tract. The patient was instructed on urinary storage and adjustments were made by drinking and urinating. Every day before irradiation, the interior of the pelvis was confirmed using ultrasound and CT.

#### CyberKnife

As a pre-treatment, all patients underwent ultrasound-guided placement of three gold fiducial markers for daily imaging guidance. Only two patients were injected with periprostatic hydrogel spacers (SpaceOAR; Augmenix, Inc., Bedford, MA), but they were not included in the final analysis. Images of 1.25 mm thickness were used for planning CT. Clinical target volume (CTV) margins of 1 mm posteriorly and 3 mm in other dimensions were added to the GTV. However, the area that overlapped with the rectal or bladder mucosa was removed from the CTV. PTV margins of 2 mm in all directions were added to the CTV. Multiplan (Accuray Inc., Sunnyvale, CA, USA) was used as the planning system. The dose constraints have been described previously [14]. In summary, the prescribed dose was adjusted to 75–85% of the peak dose, and the PTV minimum dose was set to > 70% of the peak dose. The goal for the urethra minimum dose was > 95% and the maximum dose was < 102%. A urethral maximum dose of < 110% was permitted. During irradiation, the prostate position was checked and corrected at intervals of 20–60 s using fiducial marker tracking, and the treatment time was adjusted to ≤ 35 min.

#### VMAT

None of the patients underwent the placement of gold fiducial markers or hydrogel spacers. Images of 2 mm thickness were used for the planning CT. Please note for convenience, CTV was used synonymously with GTV. PTV margins of 3 mm posteriorly and 6 mm in the other dimensions were added to the CTV. Eclipse (Varian Medical System, Palo Alto, CA, USA) or RayStation (RaySearch Laboratories, Stockholm, Sweden) was used as the planning system. Dose constraints were defined by modifying the Radiation Therapy Oncology Group (RTOG) 0938 protocol [11]. The maximum point dose to 0.03 cc (Dmax) of PTV was set to be < 107%. The tolerances for the PTV were D10% < 106%, D50% < 104%, and D98% > 95%. To prevent a steep dose drop in the seminal vesicle, the distal portion was also set to receive ≥ 80% dose. The dose to the bladder Dmax was set to be ≤ 105%, and the tolerances were V18% < 50%, V28% < 25%, and V32% < 16%. The dose at rectum Dmax was set to be ≤ 105%, and the tolerances were V18% < 50%, V28% < 20%, and V32% < 10%. The dose at urethral Dmax was set to be ≤ 107%, and the tolerances of penile bulb were D2% < 25 Gy and D50% < 14 Gy. The plans were designed and optimized according to two full arcs with flattening filter-free (FFF) beams. Since the irradiation time per arc was 1 min, the treatment time was approximately 2 min.

### Evaluation of outcomes

We measured the time to the event from the date of commencement of radiotherapy. “No biochemical evidence for the disease” was defined according to the Phoenix definition, as an increase of + 2 ng/mL in the absolute nadir PSA level, regardless of the time point [15]. We evaluated patient-reported outcomes based on the IPSS and Quality of Life (QOL) score. IPSS ≤ 7, 8–19, and 20–35 were defined as mildly, moderately, and severely symptomatic conditions, respectively. The scores were recorded before radiotherapy and at 1 week, 4 weeks, and 3 months after SBRT. They were recorded every 3 months thereafter for 1 year. They were also recorded 2 and 3 years later. The highest value up to 3 months was defined as the peak value of the acute-phase IPSS. Physician-recorded toxicities were assessed according to the National Cancer Institute Common Toxicity Criteria for Adverse Events (CTCAE, version 5.0). Acute toxicity was defined as treatment-related symptoms observed during or less than 3 months following radiotherapy. Patients receiving medication to improve dysuria before radiotherapy (baseline) were counted as an event if they required an increase in the medication dose and/or additional procedures. Late toxicity was defined as any event that persisted for 3 months or thereafter following radiotherapy.

### Statistical analyses

Continuous variables are presented as mean ± standard deviation or median values with ranges. All statistical analyses were performed using EZR version 1.55 (Saitama Medical Center, Jichi Medical University, Saitama, Japan), based on R and R commanders [16]. Comparisons of categorical variables were performed using the Fisher exact test, and comparisons of continuous variables were performed using the Student’s t-test or Mann–Whitney U test. To reduce selection bias, we performed propensity score matching analysis between the CyberKnife and VMAT groups. Propensity scores were estimated from a logistic regression model by including variables that could potentially affect GU toxicity, such as age, pre-treatment IPSS, and prostate volume. The caliper width was set at 0.2 multiplied by the standard deviation.

Using these propensity scores, patients in the CyberKnife group were matched in a 1:1 ratio with those in the VMAT group.

The transitions in patient-reported outcomes were analyzed using repeated-measures one-way analysis of variance. The mean scores at each time point were compared between the CyberKnife and VMAT groups.

We conducted univariate analysis to determine the factors that contributed to the peak value of acute-phase IPSS using single regression analysis. Factors that deviated too far from a normal distribution even after log-transformation were excluded; however, factors considered important in previous studies were converted to nominal variables. Statistical significance was set at p < 0.05. Factors demonstrating a difference (p-values < 0.1) in the univariate analysis were included in the multivariate analysis. We conducted multiple regression analysis for multivariate analysis.

The Kaplan–Meier method was used to estimate the cumulative incidence of toxicities.

## Results

### Patient characteristics and clinical outcomes

**Fig. 1:**
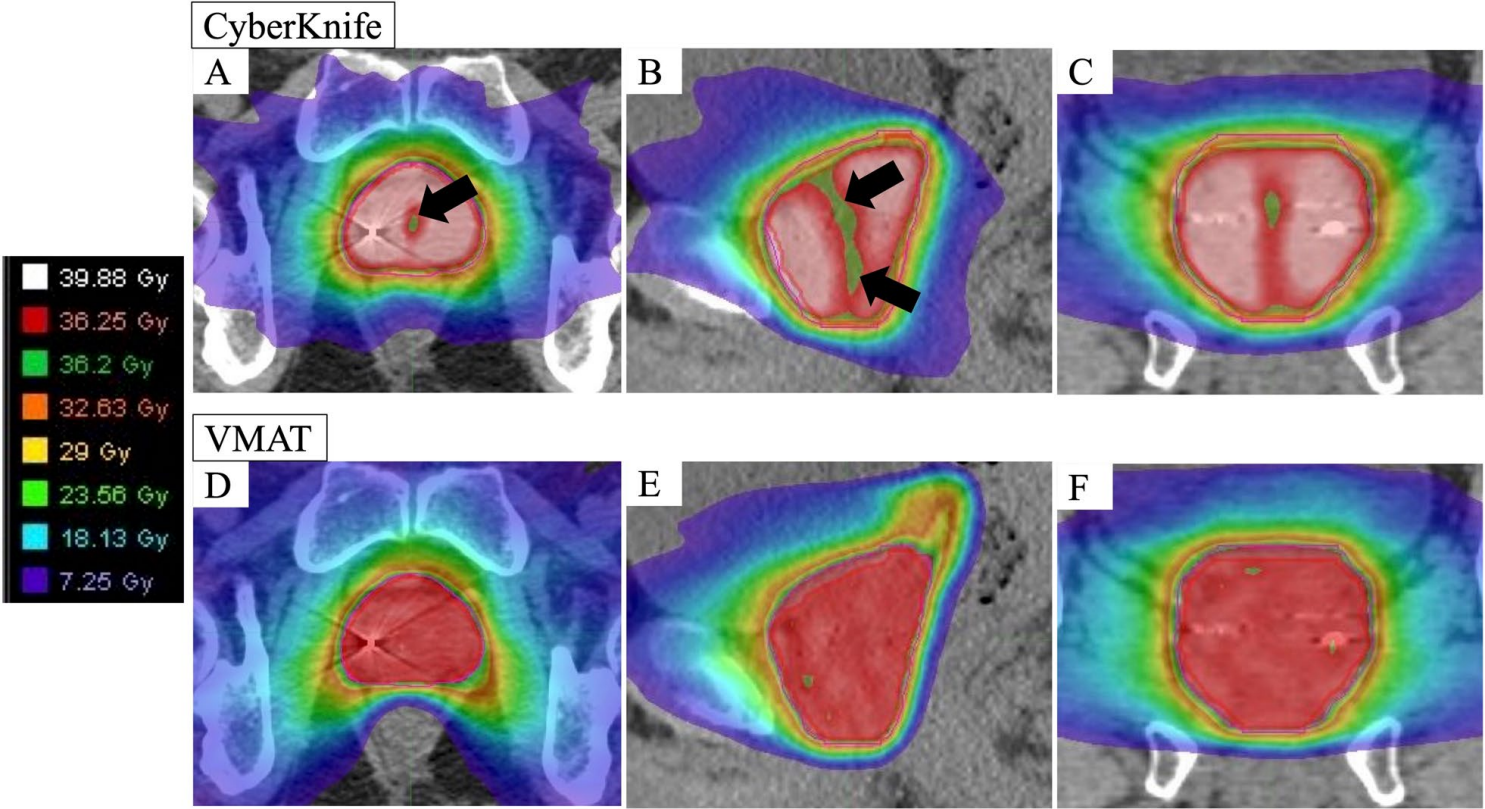
Example of dose distribution comparison between CyberKnife and VMAT. The upper row shows axial, sagittal, and coronal view of CyberKnife, starting from the left side. The bottom row shows axial, sagittal, and coronal view of VMAT from the left side. Black arrow: CyberKnife moderately reduces urethral dose. *VMAT* volumetric-modulated arc therapy

Table 1 summarizes the patient characteristics before matching. Patients in the CyberKnife group were significantly younger than those in the VMAT group. The mean age in the CyberKnife group was 68.9 ± 7.3 years, while that in the VMAT group was 72.5 ± 6.1 years (p = 0.0069). Patient and dosimetry characteristics for the 70 participants after propensity score matching are shown in Table 2. After adjusting for age bias in both the groups the values were 71.7 ± 6.3 years for CyberKnife and 71.8 ± 5.8 years for VMAT (p = 0.953). Other factors contributing to GU toxicity, namely, pre-treatment IPSS and prostate volume did not differ significantly between the two groups. In contrast, the mean PTV volume was approximately 15 cc smaller in the CyberKnife group than in the VMAT group (p = 0.002). An example of dose distribution is shown in Fig. 1; identical cases have been used for comparison.

**Table 1:** Patient characteristics prior matching

Parameter	CyberKnife (n = 104)	VMAT (n = 38)	*p* Value
Age (years)	68.9 ± 7.3	72.5 ± 6.1	0.0069
*Risk group*			0.72
Low	10 (10%)	4 (11%)	
Intermediate	60 (57%)	19 (50%)	
High	34 (33%)	15 (40%)	
Neoadjuvant ADT	95 (91%)	34 (90%)	0.75
Duration of neoadjuvant ADT (months)	8.3 ± 11.1	6.3 ± 0.9	0.29
*Comorbidity*			
Antithrombotic therapy	13 (13%)	6 (16%)	0.59
Diabetes	14 (14%)	6 (16%)	0.79
Previous TURP	2 (2%)	1 (3%)	0.99
Medication to improve dysuria at baseline	17 (16.3%)	7 (18.4%)	0.8
*Pre-treatment IPSS*			0.96
Mild (0–7)	33 (32%)	13 (34%)	
Moderate (8–19)	59 (57%)	21 (55%)	
Severe (20–35)	11 (11%)	4 (11%)	
Prostate volume (cc)	25.9 ± 9.6	26.0 ± 15.3	0.97

Data are presented as number (%) or mean ± SD

*VMAT* volumetric modulated arc therapy; *ADT* androgen deprivation therapy; *TURP* transurethral resection of the prostate; *IPSS* International Prostate Symptom Score

**Table 2:** Patient and dosimetry characteristics after propensity score matching

Parameter	CyberKnife (n = 35)	VMAT (n = 35)	*p* Value
Age (years)	71.7 ± 6.3	71.8 ± 5.8	0.953
*Clinical T stage*			0.50
1c-2a	14 (40%)	18 (51%)	
2b-2c	14 (40%)	9 (26%)	
3a	7 (20%)	8 (23%)	
*IPSA (ng/mL)*			0.70
< 10	20 (57%)	23 (66%)	
10–20	10 (29%)	7 (20%)	
> 20	5 (14%)	5 (14%)	
*Gleason score*			0.775
6	3 (8%)	5 (14%)	
7	24 (69%)	21 (60%)	
8–10	8 (23%)	9 (26%)	
*Risk group*			0.82
Low	2 (5.7%)	4 (11.4%)	
Intermediate	19 (54.3%)	17 (48.6%)	
High	14 (40%)	14 (40%)	
Neoadjuvant ADT	33 (94.3%)	31 (88.6%)	0.673
Duration of neoadjuvant ADT (months)	6.4 ± 3.2	6.3 ± 0.8	0.867
*Comorbidity*			
Antithrombotic therapy	5 (14.3%)	6 (17.1%)	0.99
Diabetes	5 (14.3%)	4 (11.4%)	0.99
Previous TURP	0 (0%)	1 (2.9%)	0.99
Medication to improve dysuria at baseline	7 (20%)	7 (20%)	0.99
*Pre-treatment IPSS*			0.939
Mild (0–7)	10 (28.6%)	12 (34.3%)	
Moderate (8–19)	21 (60.0%)	19 (54.3%)	
Severe (20–35)	4 (11.4%)	4 (11.4%)	
Median follow-up (years)	3.0 (1.1–4.6)	2.9 (1.6–4.2)	0.36
Prostate volume (cc)	25.3 ± 9.3	25.3 ± 15.5	0.997
PTV volume (cc)	47.3 ± 13.0	62.4 ± 23.9	0.002
PTV D98% (Gy)	35.4 ± 0.30	35.8 ± 0.15	< 0.001
PTV Dmedian (Gy)	41.8 ± 0.76	37.1 ± 0.20	< 0.001
PTV D2% (Gy)	44.0 ± 0.80	37.7 ± 0.24	< 0.001
Bladder volume (cc)	191.0 ± 44.8	237.5 ± 102.5	0.0165
Bladder Dmean (Gy)	9.75 ± 1.64	9.74 ± 2.43	0.979
Bladder D0.5 cc (Gy)	39.7 ± 1.10	37.5 ± 0.18	< 0.001
Bladder Dmax (Gy)	41.9 ± 1.40	37.8 ± 0.19	< 0.001
Urethra Dmean (Gy)	36.0 ± 0.18	37.1 ± 0.30	< 0.001
Urethra D0.1 cc (Gy)	36.2 ± 0.22	37.4 ± 0.37	< 0.001
Urethra Dmax (Gy)	37.1 ± 0.40	37.5 ± 0.40	0.002
Rectal Dmean (Gy)	9.80 ± 1.75	9.80 ± 1.76	0.99
Rectal D0.5 cc (Gy)	35.6 ± 1.45	36.3 ± 1.31	0.034
Rectal Dmax (Gy)	38.9 ± 0.89	37.4 ± 0.50	< 0.001

Data are presented as median (range), mean ± SD, or number (%)

*VMAT* volumetric modulated arc therapy; *IPSA* initial prostate-specific antigen; *ADT* androgen deprivation therapy; *TURP* transurethral resection of the prostate; *IPSS* International Prostate Symptom Score; *PTV* planning target volume; *Dmedian* median dose; *Dx%* dose covering x% of the target volume; *Dmean* mean dose; *Dx cc* dose to x cc of the organ; *Dmax* maximum dose

Dmedian and D2% of PTV were significantly higher in the CyberKnife group, but urethral dose was significantly lower in the CyberKnife group than in the VMAT group.

With a median follow-up time of 3 years in both the groups, there were no cases of biochemical/clinical recurrence, distant metastasis, or death from prostate cancer. In the CyberKnife group, only one patient died of a cause other than prostate cancer. This patient developed myelodysplastic syndrome 1.5 years after radiotherapy and died 7 months later. However, the causal relationship with radiotherapy remains unclear.

### Patient-reported outcomes

**Fig. 2:**
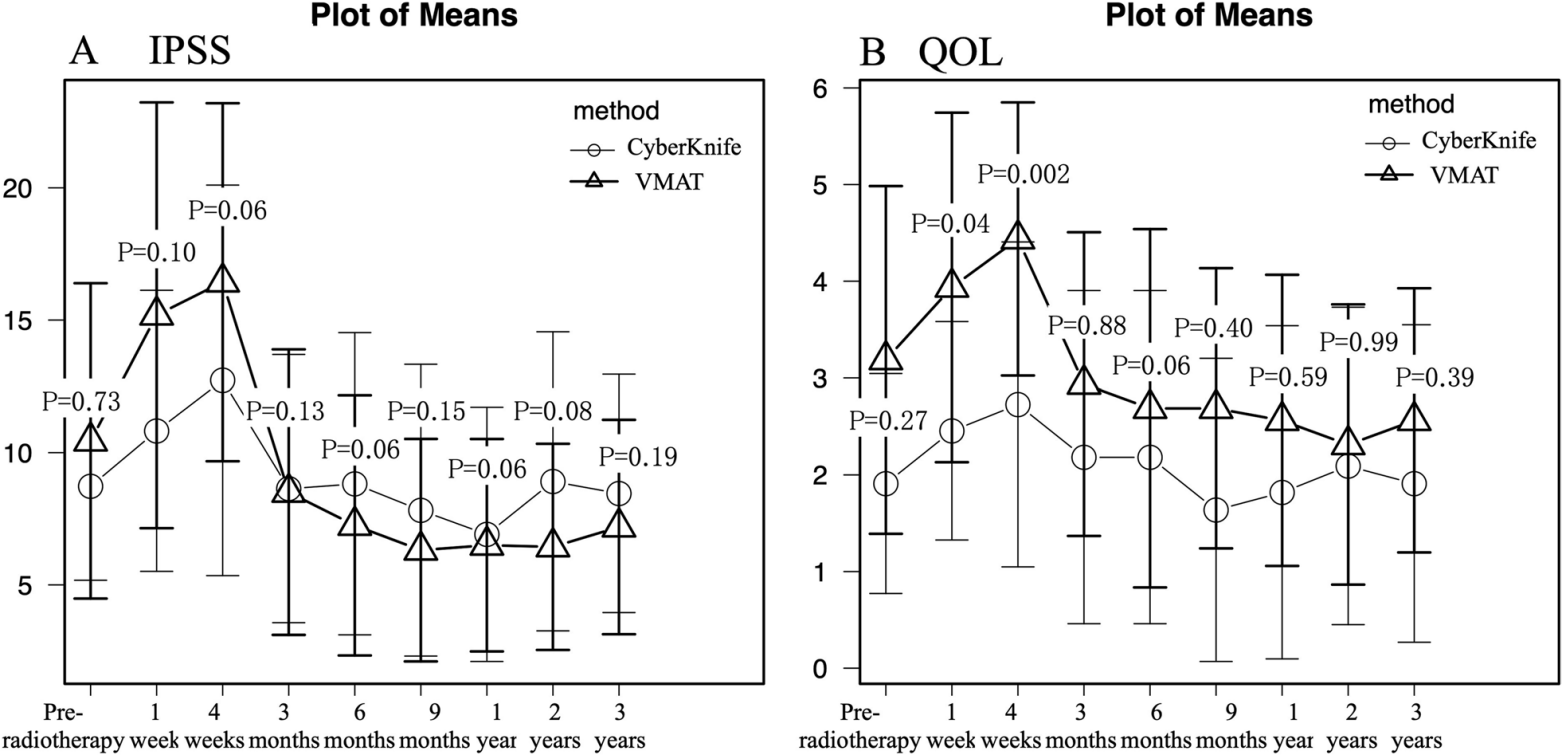
Transitions in the IPSS **A** and QOL score **B** following radiotherapy. Each p-value indicates a comparison between modalities. *IPSS* International Prostate Symptom Score; *QOL* quality of life; *VMAT* volumetric-modulated arc therapy

Figure 2A shows the transitions in the IPSS. Regardless of the treatment modality, scores peaked at 4 weeks and returned to baseline values by 3 months. The scores of the CyberKnife group tended to be lower than the VMAT group at 1 and 4 weeks. The peak values in acute phase of IPSS were significantly lower in the CyberKnife group (CyberKnife: 16.2 ± 7.5 vs VMAT: 20.2 ± 7.1, p = 0.025) than in the VMAT group. The results of the univariate and multivariate analyses of IPSS peak values are shown in Table 3. The treatment modality (CyberKnife vs. VMAT) independently contributed to the peak value of acute-phase IPSS (*p* = 0.03). PTV volume was divided by the median (49.5 cc) and a larger PTV volume (> 49.5 cc) had a significant effect on the peak values of IPSS in the univariate regression analysis (*p* = 0.004). Three other factors, age (*p* = 0.01), medication to improve dysuria at baseline (*p* = 0.03), and neoadjuvant ADT (*p* = 0.04) also contributed to the peak value of acute-phase IPSS in the multivariate regression analysis.

**Table 3:** Univariate and multivariate analyses of the peak values in acute phase of IPSS

Explanatory variable	Single regression				Multiple regression		
	Parameter estimates	Standard error	95% CI	*p* Value	Parameter estimates	Standard error	*p* Value
Age (years)	0.38	0.14	0.1–0.67	0.01	0.35	0.13	0.01
Risk group	−2.06	1.43	−4.92–0.8	0.16			
Neoadjuvant ADT	−6.01	3.15	−12.2–0.28	0.06	−5.88	2.85	0.04
Antithrombotic therapy	0.34	2.49	−4.63–5.3	0.89			
Diabetes	−5.8	2.62	−11.0–0.6	0.03	−4.25	2.36	0.08
Medication to improve dysuria at baseline	5.05	2.18	0.7–9.4	0.023	4.43	2.01	0.03
Treatment modality	4.0	1.75	0.52–7.48	0.024	3.63	1.60	0.03
Larger PTV volume (> 49.5 cc)	5.09	1.70	1.69–8.48	0.004	1.99	1.74	0.25
Bladder volume (cc)	−3.03	6.34	−15.7–9.6	0.63			
Bladder Dmean (Gy)	0.44	0.44	−0.44–1.32	0.32			
Urethra Dmean (Gy)	225.6	123.1	−19.9–471.2	0.07	−13.9	282.0	0.96
Urethra Dmax (Gy)	1.42	2.11	−2.78–5.62	0.50			

*IPSS* International Prostate Symptom Score; *CI* confidence interval; *ADT* androgen deprivation therapy; *PTV* planning target volume; *Dmean* mean dose; *Dmax* maximum dose

The QOL scores presented in Fig. 2B showed a trend similar to that of the IPSS. QOL scores after 1 week (CyberKnife: 3.1 ± 1.8 vs VMAT: 4.0 ± 1.6, *p* = 0.04), 4 weeks (CyberKnife: 3.3 ± 1.6 vs VMAT: 4.4 ± 1.3, *p* = 0.002), and peak values of QOL scores (CyberKnife: 3.7 ± 1.6 vs VMAT: 4.7 ± 1.3, *p* = 0.01) were all significantly lower in the CyberKnife group. However, the pre-radiotherapy QOL scores did not match between the two groups. When analyzed by the amount of change in values from pre-radiotherapy QOL score, there were no differences between groups at 1 week (p = 0.19), 4 weeks (*p* = 0.14), or in their peak values (*p* = 0.17).

### Physician-recorded toxicities

**Fig. 3:**
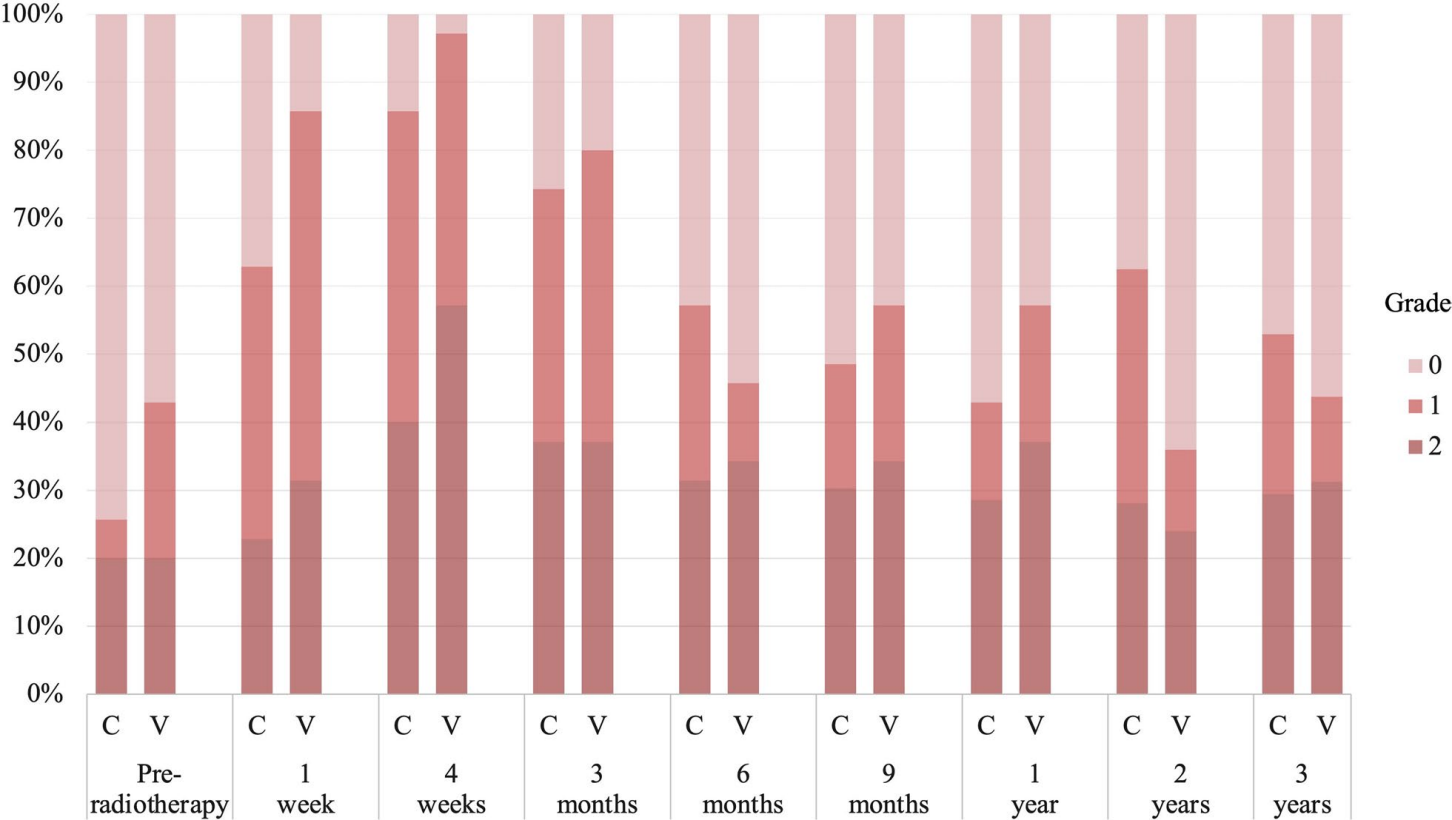
Genitourinary toxicity grades according to timepoint. *C* CyberKnife; *V* volumetric-modulated arc therapy

**Fig. 4:**
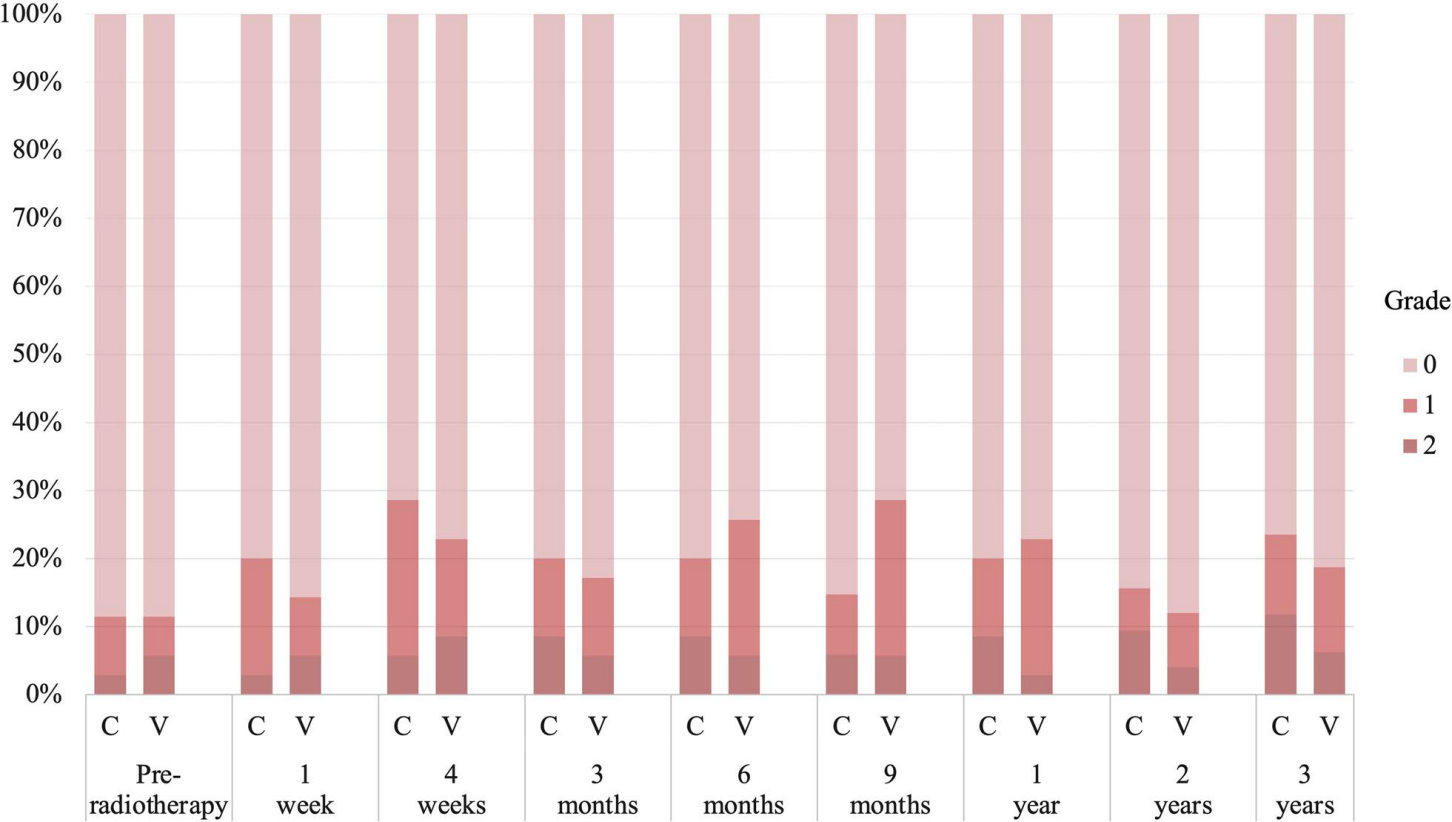
Gastrointestinal toxicity grades according to timepoint. *C* CyberKnife; *V* volumetric-modulated arc therapy

No grade 3 or higher toxicity was observed in any category. The incidence of treatment-related grade 2 acute GU toxicity tended to be lower in the CyberKnife group (CyberKnife: 8 [22.9%] vs. VMAT: 16 [45.7%], *p* = 0.077) than in the VMAT group. The frequently identified categories (including duplicates) were grade 2 urinary retention in 5 patients and urinary frequency in 3 patients in the CyberKnife group, and grade 2 urinary retention in 8 patients and urinary frequency in 9 patients in the VMAT group. No difference was noted between groups in cumulative incidence of treatment-related grade 2 late GU toxicity at 3 years (CyberKnife: 35.3% [20.8–55.6%] vs. VMAT: 25.7% [14.3–43.6%], *p* = 0.67). The GU toxicity grades according to the time point are shown in Fig. 3. There was no significant difference between the two groups in terms of the GU grade at any time point. Treatment-related grade 2 acute GI was observed in two patients (5.7%) with constipation in the CyberKnife group and one patient (2.9%) with diarrhea in the VMAT group, but the incidence did not differ. No difference was noted between groups in cumulative incidence of treatment-related grade 2 late GI toxicity at 3 years (CyberKnife: 8.6% [2.8–24.3%] vs. VMAT: 5.7% [1.5–21.0%], p = 0.65). There were no significant differences in the GI toxicity grades between the two groups at any time point up to 3 years (Fig. 4). Other grade 2 toxicities included erectile dysfunction in one patient (3%) in the CyberKnife group and gynecomastia in one patient (3%) in the VMAT group.

## Discussion

To the best of our knowledge, this is the first report to analyze SBRT outcomes for prostate cancer by treatment modality adjusted for patient background using propensity score matching. In a total of 70 patients selected from 142 patients, the clinical outcome was good, and no serious toxicity was observed regardless of the modality. Based on patient-reported outcomes, CyberKnife has a slight advantage over VMAT in terms of acute prostate symptoms.

Only one previous high-quality study has focused on the toxicity of each treatment modality. PACE-B is an international, phase 3, open-label, randomized trial aimed at assessing the non-inferiority of SBRT compared to conventionally fractionated or moderately hypofractionated radiotherapy for prostate cancer [4]. A subset analysis examining acute toxicity reported RTOG grade 2 or more severe GU toxic effects for patients treated using non-CyberKnife (75 [31%] of 245 patients) versus those treated using CyberKnife (21 [12%] of 170 patients) delivery were significantly different (difference: -18.3 percentage points, 95% confidence interval: −10.7 to −25.9; *p* < 0.0001). Although the methods of analysis were different, the real-world data we have presented support this earlier report. Recently, an additional report of the PACE-B trial at 2 years was published [17].

According to this report, CTCAE GU grade 2 or worse toxicity at 2 years was less frequent in patients treated with CyberKnife than in those treated with non-CyberKnife (9 [6%] of 154 patients vs. 35 [17%] of 212 patients; p = 0.0020). However, there was no difference in the RTOG grade, and it was believed that disparities between centers and fiducial markers may have been the confounding factors. We believe that in our study the long-term follow-up of patients was insufficient, and further studies are required to assess late toxicity.

We were unable to identify factors by which CyberKnife reduced acute prostate symptoms. However, margin setting may be the most important factor. Larger PTV volume had a significant effect on the peak values of IPSS in the univariate regression analysis (*p* = 0.004). The reported increase in vicinity of the membranous, spongy urethra and urinary trigone doses due to PTV expansion is associated with GU toxicity [18, 19], supporting our findings. CyberKnife is advantageous in reducing margins because fiducial marker tracking is accurate to less than 1 mm, while image guidance in VMAT is visual and requires consideration of human error. As the MIRAGE study recently showed, minimizing margins by using MRI guidance may be the most effective strategy for reducing GU toxicity in the future [20]. Moreover, the urethral dose may be another factor. Urethra Dmean tended to contribute to the peak values in the acute phase of IPSS in univariate analysis (*p* = 0.07). Several studies have suggested that urethral dose contributes to GU toxicity [21, 22]. Although it is theoretically possible to reduce the urethral dose even with VMAT, it is impossible to form a dose gradient as steep as that formed with CyberKnife. A randomized phase 2 trial that attempted to reduce the urethral dose with intensity modulation techniques warned that the rate of PSA failure was higher than that with standard therapy [23]. The ability to deliver high doses to other prostate tissue, while moderately reducing the dose to the urethra is a major advantage of CyberKnife. There are many other factors between CyberKnife and VMAT that we have not been able to examine, such as margin setting, treatment time, and image-guided accuracy. Furthermore, acute GU toxicity is complicated by multiple factors and may be difficult to analyze. However, in this study, the analysis was adjusted for patient background contributing to GU symptoms, including age, pre-treatment IPSS, and prostate volume. We believe that the factor that caused the difference in acute prostate symptoms was the treatment intervention and, broadly speaking, the treatment modality.

We emphasize that this report does not negate the use of SBRT for prostate cancer with VMAT. The efficacy and safety of SBRT using the VMAT technique has already been reported [10]. In addition, VMAT (especially with FFF beam) has a shorter treatment time than CyberKnife [19]. VMAT can be performed without fiducial markers using cone-beam CT and other matching techniques [24]. These are significant advantages for patients who desire minimally invasive treatment and are sufficient reasons to perform SBRT with VMAT, regardless of whether the institution owns a CyberKnife.

Our study was limited by its nonrandomized retrospective nature and a small sample size. GI toxicity has fewer events than GU toxicity and requires a larger sample size for accurate group comparisons. Moreover, a longer follow-up is required to assess treatment efficacy and late toxicities. We intend to accumulate the aforementioned data for future research.

## Conclusions

Regardless of the treatment modality, SBRT for prostate cancer has shown good 3-year treatment efficacy without serious toxicity. Patient reports indicated that CyberKnife can significantly reduce peak prostate symptoms compared to VMAT. Physician-reported GU toxicity results showed a similar trend, supporting the slight advantage of CyberKnife. Further research with additional data and longer follow-up is required to determine the differences in GI toxicity and treatment efficacy between modalities.

## Data Availability

Research data are stored in an institutional repository and shared upon request to the corresponding author.

## Abbreviations

SBRT-related: Stereotactic body radiation therapy-related
GU: Genitourinary
IPSS: International Prostate Symptom Score
VMAT: Volumetric-modulated arc therapy
MRI: Magnetic resonance imaging
PSA: Prostate-specific antigen
ADT: Androgen deprivation therapy
CT: Computed tomography
GTV: Gross tumor volume
PTV: Planning target volume
GI: Gastrointestinal
CTV: Clinical target volume
RTOG: Radiation therapy oncology group
FFF: Flattening filter-free
QOL: Quality of life
IPSA: Initial prostate-specific antigen
TURP: Transurethral resection of prostate
CI: Confidence interval
